# Congenital Neonatal Intestinal Obstruction

**DOI:** 10.21699/jns.v5i4.472

**Published:** 2016-10-10

**Authors:** Ashrarur Rahman Mitul

**Affiliations:** Professor, Pediatric Surgery at Dhaka Shishu(Children) Hospital, Bangladesh

Intestinal obstructions among neonates are almost always congenital and pose a huge challenge for the neonatal as well as for the pediatric surgeons globally. The different physiological features from older children place all neonates in a unique group of population. Neonates with intestinal obstructions constitute major cause of surgical admissions in all pediatric hospitals. In a very superficial review, till the latest issue of the 5th year of publication of this indexed journal, a total of approximately 45 articles were focused on some kind of congenital neonatal obstructions of different etiologies. Such is the magnitude of the problem, the reason why the editorial board took the noble decision to bring out an special issue addressing this particular group of anomalies which almost always present in the same way clinically and and the management objectives basically remain the same, to relieve the obstruction after adequate resuscitation, which almost always is a demanding task. 

A wide variety of anomalies are responsible for congenital intestinal obstruction in neonates. Artesia and stenosis at different levels of intestine, annular pancreas, malrotation of gut with its different spectrums, meconium diseases, Hirschsprung disease and Anorectal malformations of varied presentations are among the commonest etiologies. Congenital short left colon syndrome, bands and adhesions, Megacystis microcolon intestinal hypoperistalsis syndrome and others constitute the rarer causes of obstruction. However some kind of incomplete obstructions may not present acutely in the neonatal period, and may be brought to attention at an older age e.g. stenosis, membranes with central hole or malrotation. Different respected authors will try to share their valuable experiences on this subject in this special issue.

Bilious vomiting, abdominal distention and failure to pass meconium or stool are the more prominent features of presentation in most cases. Of course the sequence of appearance of symptoms varies depending on the level of obstructed bowel. Bilious vomiting from the very outset or soon followed by a few episodes of non-bilious ones, should always be considered significant and surgical unless proved otherwise. Almost all term healthy neonates should pass meconium within first 24 hours of life; any delay in the passage of meconium beyond 24 hours should raise the suspicion of obstruction, if no co-morbid medical conditions like hypothyroidism or sepsis are suspected. There may be few exceptions like Hirschsprung's disease, meconium ileus and the early stages of volvulus when a small amount may be passed initially. Abdominal distension however may not be prominent in upper gut obstruction although epigastric fullness may be observed. Bilious vomiting with per rectal bleeding is an ominous scenario and indicates bowel ischemia as in volvulus neonatorum. Babies may even present with complications like gut perforation or Necrotizing Enterocolitis (NEC) with shiny, hugely distended, tense abdomen. The most alarming scenarios among neonates with gut obstruction are that they deteriorate rapidly due to early appearance of shock, electrolyte imbalance, acidosis and sepsis because of their unique hemodynamic and physiological status unless presented or attended early. Furthermore, presence of associated anomalies like cardiac, renal or metabolic makes them even more susceptible to the derangements caused by the bowel obstructions. Down syndrome with duodenal atresia, cystic fibrosis and meconium ileus, Anorectal Malformations and VACTERL are well established associations and should always be looked for. 

Early diagnosis is of utmost importance in the successful management of these babies. Antenatal screening with ultrasonography may show dilated bowel loops; maternal polyhydramnios might give a suspicion to an upper gut obstruction. The importance of proper history including timing of passage of meconium, vomiting, if present, with its characteristics, and clinical examinations including a full abdominal and complete perineal examination are of paramount importance. The most informative post natal investigation is a plain abdominal X-ray; double-bubble of duodenal obstruction, soap bubble appearance in meconium ileus, calcifications of prenatal bowel perforations are some of the finding which are suggestive. In some cases a water soluble contrast x-ray may become essential the (I personally feel Barium should be avoided among the neonates because of the complications associated with its use). USG may be helpful in some cases too. 

Transport of neonates with bowel obstruction should be meticulous because tiny kids tolerate handling and movements poorly. Emergency transport system should be involved wherever possible; however this is a far cry in most of the resource constraint places, a factor that negatively influence the outcome ultimately. Management of neonatal congenital obstruction are almost always surgical with very few exceptions like meconium ileus, meconium plug or short left colon syndromes where an initial non-operative may be tried and may be successful. But whatever is the case, there should never be any haste to rush the baby to the theatre without adequate resuscitative measures been undertaken, otherwise the mortality would be high. Measures including keeping the baby nothing per oral, N/G suction, polyelectrolyte and dextrose containing intravenous fluids and of course broad spectrum antibiotics should be initiated in a euthermic environment; blood glucose, electrolytes and acid base statuses should be monitored and corrected regularly preferably in a NICU set up. Neonatal surgeons or properly trained pediatric surgeons should deal with these cases for satisfactory outcome. Post operative parenteral nutrition should be part of the management protocol, the lack of which will severely negate the survival.

Challenges and difficulties will be there in every aspect of medical management, particularly when a surgical neonate with congenital obstruction is concerned. There comes the importance of cooperation among neonatal and pediatric surgeons worldwide. Exchange of knowledge among the peers within a particular geopolitical region and beyond are of paramount importance for improvement in this area. Sharing of experiences, difficulties in management and their solutions wherever they are available should get highest priorities. Every human brain is a unique inventor; and it is true that some surgeons may be handling a situation in an effective otherwise unknown way utilizing locally available facilities which may be shared with others. So publication of rare cases, difficulties faced in a particular previously unidentified situation or the way the problem was solved may all be the areas to be published. Availability of print and electronic media, social networks, neonatal or pediatric surgical groups are available so widely during these days of information technology. National coordination and international cooperation are essential to face the challenge. It is the reality that discrimination of wealth do persist among different regions of the world but collaboration among physicians dealing with these surgical neonates may overcome these barriers if true noble intensions exist and ways out may be explored. Child mortality indices heavily depend on neonatal mortality and surgical neonates must get highest priority because of the unique kind of its nature to fight the battle; otherwise all efforts will go in vein ultimately. 

This has been an awesome experience on my part to work as the guest editor for this special issue. I remain thankful to the editorial board of the journal who believed in me to assign the huge responsibility. The unique system followed by the open journal system (OJS) has been very tedious though, been a great lesson for me, and will remain as a milestone of experiences on my part. The cooperation I received from the members of the editorial board was incredible and unforgettable. Not mentioning names, some of these friends have simply amazed me as a whole with their capabilities. The response from different authors has been overwhelming to be very frank. But, I must admit, I have definite limitations and I apologize for anything that falls short of everyone's expectations. 

The entire editorial board expresses its whole hearted gratitude to all the authors for their contribution to this special issue. Each and every one of the respected reviewers needs particular thanks for spending their valuable time to evaluate the manuscripts minutely for a quality output. Lastly, everyone who was involved in the different stages of the publication of this special issue deserves heartfelt felicitations without which this work would never be possible.

## Footnotes

**Source of Support:** Nil

**Conflict of Interest:** The author is editor of the journal. This article is not externally peer reviewed.

